# Stigma and Smoking in the Home: Parents’ Accounts of Using Nicotine Replacement Therapy to Protect Their Children from Second-Hand Smoke

**DOI:** 10.3390/ijerph17124345

**Published:** 2020-06-17

**Authors:** Grace Lewis, Neneh Rowa-Dewar, Rachel O’Donnell

**Affiliations:** 1 School of Healthcare, University of Leeds, Leeds LS2 9JT, UK; 2 USHER Institute, University of Edinburgh, Edinburgh, H8 9AG, UK; neneh.rowa-dewar@ed.ac.uk; 3 Institute for Social Marketing and Health, University of Stirling, Stirling, FK9 4LA, UK; r.c.odonnell@stir.ac.uk

**Keywords:** stigma, nicotine replacement therapy, second-hand smoke, smoke-free homes, health inequality, qualitative, parents, socioeconomic disadvantage

## Abstract

Evidence and campaigns highlighting smoking and second-hand smoke risks have significantly reduced smoking prevalence and denormalised smoking in the home in Scotland. However, smoking prevalence remains disproportionally high in socioeconomically disadvantaged groups. Using stigma as a theoretical lens, this article presents a thematic analysis of parents’ accounts of attempting to abstain from smoking at home, using nicotine replacement therapy (NRT), in disadvantaged areas of Edinburgh and the Lothians. Smoking stigma, particularly self-stigma, underpinned accounts, with two overarching themes: interplaying barriers and enablers for creation of a smoke-free home and reconceptualisation of the study as an opportunity to quit smoking. Personal motivation to abstain or stop smoking empowered participants to reduce or quit smoking to resist stigma. For those struggling to believe in their ability to stop smoking, stigma led to negative self-labelling. Previously hidden smoking in the home gradually emerged in accounts, suggesting that parents may fear disclosure of smoking in the home in societies where smoking stigma exists. This study suggests that stigma may act both as an enabler and barrier in this group. Reductions in smoking in the home were dependent on self-efficacy and motivations to abstain, and stigma was entwined in these beliefs.

## 1. Introduction

Smoke-free legislation for public spaces protects over 1.6 billion people, approximately 22% of the global population, from second-hand smoke (SHS) [1]. However, many, including children, are exposed to SHS in the home. Without an accepted safe level of exposure, strategies to improve protection are required. Children are at greater risk from SHS exposure, due to physiological respiratory differences in the developing child and their immune systems [2], and their inability to protect themselves in the home [3]. Consistent and robust evidence supports links between SHS and childhood conditions including sudden infant death syndrome (SIDS), asthma and increased risks of other respiratory conditions. Many other conditions have been associated with SHS and evidence is continually evolving [4,5]. Health consequences of direct smoking are well known, and an analysis of data from the United Kingdom (UK) highlighted that Scotland had the highest proportion of cancer cases attributable to smoking in 2015 [6]. Additionally, SHS increases the risk of lung cancer by 20–30% in never-smokers [4]. SHS has been associated with childhood cognitive deficits, and further research is necessary to understand this association [7]. Associations between adolescent smoking uptake and parental smoking have been reported [8,9]. However, how parents perceive such potential risks to their children is complex and socially constructed [10].

Smoke-free legislation and campaigns in Scotland led to denormalisation of smoking and contributed to reductions in SHS exposure in the general population [11]. However, legislation does not extend into private spaces, and research has shown that denormalisation following smoke-free legislation led to increased perception of stigma amongst smokers in both the United States of America (USA) [12], and in Scotland [13]. Following legislation, there were concerns that smoking could be displaced into homes. Although this was not proven by studies in the UK [11,14,15], an increase in prevalence of childhood SHS exposure was reported in one cross-sectional study following anti-tobacco legislation in Hong Kong [16].

Despite conflicting international data over displacement, smoking prevalence has shifted predominantly to lower socioeconomic groups in recent decades [17,18,19], and this disparity was reflected in higher child SHS exposure for lower-income groups in the UK [14]. In Scotland, socioeconomically disadvantaged children remain disproportionally exposed to SHS. The World Health Organisation (WHO) describes definitions of health inequality extended to include exposures beyond individual control [20]. Young children’s SHS exposure in the home can be considered an inequality under this definition. Despite targets to reduce childhood exposure by 50% by 2020 being achieved, 15% of children in the most disadvantaged areas remain exposed, in contrast to 1% in the least disadvantaged areas of Scotland [21]. Denormalisation and the socioeconomic shift of smoking to predominantly lower classes has arguably made the stigmatisation of smoking as socially disagreeable easier for societies [22].

Stigma definitions vary, with many based upon the work of Goffman and the socially constructed “spoiled identity” accompanying stigma. Goffman defined stigma as “an attribute that is deeply discrediting”, tainting and compromising individuals [23], (pp.15). Further, Goffman described those discredited by stigma, where a stigmatising feature is outwardly visible. Conversely, where the stigmatising feature can mostly be hidden, individuals are considered to have a discreditable stigma [23]. Smoking in the home may mitigate stigma to some extent, as it can be hidden from public view. However, for those who smoke frequently, the lingering smell of smoke and resultant expected stigma [10], renders smoking potentially noticeable in societies where smoking is denormalised. Whether children living in homes with smokers are stigmatised due to smoke smell on their person or belongings is a possibility that has been less explored. Evolving definitions and stigma theory provide a means to conceptualise stigma and expand our understanding the implications of stigma. Link and Phelan [24], sought to address critiques that existing stigma definitions were vague and “individually focused” (p.363), by reconceptualising stigma as interconnected elements: labelling, stereotyping, separation, status loss and discrimination. Link and Phelan also acknowledged that “stigma exists as a matter of degree” [24], (pp.376). This may suggest that even those with a stigmatised status that can be predominantly concealed from public view, may still anticipate stigma, and feel anxiety at the idea their smoking may be revealed.

Although many stigma theories exist following research into specific health conditions, some conceptualisations can be adapted to discuss smoking stigma. Pryor and Reeder’s [25], conceptual model was adapted from their work in the field of HIV and stigma [26]. The model encompasses four interlinked stigma sources, with public stigma central and self-stigma, stigma by association and structural stigma (the latter describes sociopolitical, legislative or institutional enactment) as an interlinked stigmatising cycle [26,27]. This model can be applied to smoking in the UK context, since anti-smoking campaigns and legislation (structural), led to public denormalisation and arguably smoking stigma. Our analysis will highlight how self-stigma and anticipated stigma by association can follow.

Existing literature exploring stigma experienced by parents attempting to create smoke-free homes (SFH) is sparse. A systematic synthesis of barriers and enablers for creating SFHs highlighted the need to balance multi-factorial issues affecting decisions and practicalities in working towards a SFH. Avoiding stigma was viewed as a motivator when participants wished to avoid smoke smell, guilt associated with smoking or being viewed by others as irresponsible for exposing children to smoke. However, the authors acknowledged that although a motivator for some, further stigmatisation of parents who are already attempting to do the best they can manage, in their often complicated circumstances, should be avoided [28].

There is a clear ethical necessity to protect children from SHS in the home [29]. However, for parents and carers with young children and limited direct access to outdoor space, there are limited options for smoking privately to avoid anticipated judgement, whilst balancing caring for young children.

Nicotine is recognised as addictive and is therefore a strong factor influencing smoking continuation. Evidence suggests that smokers on lower incomes have a higher nicotine intake, due to inhalation technique and/or quantity smoked, and therefore higher nicotine dependency in some cases [18]. The 2014 harm-reduction addendum to Scottish National Institute for Health and Care Excellence guidelines recommended that smoking cessation services should consider prescribing (or signposting to providers and advisors) nicotine replacement therapy (NRT) for temporary smoking abstinence to manage nicotine cravings and reduce SHS exposure for others, particularly where smoking outside is constrained by living circumstances [30].

The participants we describe are from early recruitment into a feasibility study to explore NRT use for smoking abstinence to protect children from SHS in the family home, and the methods and wider findings of the feasibility study will be reported elsewhere [31].

## 2. Materials and Methods

### 2.1. Participants

Participants were purposively sampled, based on context [32,33], due to their difficulty smoking outside, owing to outdoor access constraints and/or increasingly mobile young children, as shown in previous research [34,35,36]. For the purposes of this analysis, seven participants were sequentially selected for inclusion from the wider feasibility assessment of the harm-reduction strategy [31].

### 2.2. The Harm-Reduction Strategy

Prior to recruitment, potential participants were provided with information packs during visits to their local early years centre (EYC). Parents are referred to EYCs by social services for community support when experiencing vulnerability or challenging life circumstances. All participants were recruited at EYCs with the help of staff or through family nurse practitioners, who are familiar with family circumstances and provide necessary support and liaison with social services. All EYCs were in areas considered disadvantaged under the Scottish Index of Multiple Deprivation (SIMD), [37]. Smoking cessation professionals later visited the EYCs to provide NRT advice and support to those interested in participating. Those who decided to participate were signposted to visit a local participating community pharmacy to collect their free NRT supply. This was followed by invitations to be interviewed after trying NRT at home.

### 2.3. Data Collection Methods

Interviews were conducted between November 2018 and February 2019 and lasted 45 min to an hour. Interviews were arranged for twelve weeks after NRT was prescribed, or earlier if:

1. Participants decided against using or continuing with NRT for the full twelve weeks (but consented to being interviewed about the experience)
2. Participants had stopped smoking before the twelve-week period had ended.

The flexibility of interview timing actualised the aim of capturing true participant experiences and reflected the ethical rights to withdraw at any time fully or partially from the study [38]. Interviews were digitally recorded with informed verbal and written consent.

Interview settings were at the preference of participants; some took place in participants’ homes and some at the EYCs, where a familiar childcare option was offered. Four participants preferred to be interviewed in pairs. Two were distant family relatives and two were a couple. Paired interviews presented initial difficulties with transcription, with fast and overlapping speech. These were overcome by a second member of the research team checking transcripts against recordings. Pre-piloted semi-structured interview guides enabled naturalistic conversational flow, whilst building rapport [39] (the interview guide is available upon request). These aimed to draw out experiences of participants’ social worlds [40], whilst providing some structure to cover broadly similar topics with all participants, with flexibility to uncover unanticipated topics [41].

### 2.4. Data Analysis

The digitally recorded interviews were transcribed by one member of the research team (GL), alongside interviewer (RO) field notes. Transcripts were anonymised and pseudonyms replaced participant names. Transcription formed the foundation of early repeated analysis, with care taken to transcribe verbatim, including pauses, laughter, changes in tone or pace. This is considered most ethical in avoiding misrepresentation [42], and best practice to protect against replication of researcher preconceptions [43]. Thematic analysis was guided by analytic steps outlined by Braun and Clarke [44,45], and Braun, Clarke and Rance [41], which provided a flexible and iterative analytical approach. Analysis was inductive, aiming to work from the data up [45]. This involved extensive memo trails and reflexivity to surface preconceptions, through journaling, and research team discussion [46]. The research team discussed their positionality throughout the research process. As a team we felt able to balance both never-smoker and former smoker statuses, presenting us with a balanced “insider-outsider” perspective [47], (pp55). The interviewer’s ability to develop rapport permitted openness and led participants to gradually expand on earlier accounts of smoking practices. We considered this important where participants may have feared disclosing smoking practices. Arguably, where researchers do not have direct, personal experience of context, such as social disadvantage, participants are relied upon as experts, which can empower and aid motivation [48]. Analysis was performed with an interpretivist perspective, which holds that social interactions influence behaviour and opinions [49]. The social and individual complexities surrounding smoking practices align well with an interpretivist perspective, particularly since research describes social influences in smoking decisions and practices [50]. Initial code and early theme generation was broad and reflected the richness of the data. Stigma, in different guises, was the strongest feature and presented a new lens with which to view the data. Final analysis stages were made alongside stigma theory.

Ethical approval was granted by the USHER Institute Ethics Group (UREG), University of Edinburgh (1706). Participants were offered a fifteen pounds sterling shopping voucher to recognise study participation.

## 3. Results

### 3.1. Participant Demographics

Six mothers and one father were interviewed. Parents’ ages ranged from to 18 to 39. The number of children in the home ranged from one to six, and their ages ranged from six months to sixteen years. Four participants were single parents. Families recruited were considered disadvantaged, both socioeconomically and due to additional features that may put them at a social disadvantage, such as history of homelessness, unemployment, mental health diagnoses, lone parenting, chronic health conditions, parenting children with behavioural conditions, partner absence through incarceration and lack of access to private outdoor space.

All participants smoked cigarettes at the point of recruitment, with some smoking in the home, either in a doorway or in a selected room, struggling to balance minimising child smoke exposure with child supervision. The aim of the harm-reduction approach was smoking abstinence in the home to protect children from SHS.

### 3.2. Changes to Smoking Practices

Changes in the number of cigarettes smoked and smoking locations were evident in participant accounts and provide context for thematic findings. Five participants collected NRT. Two quit smoking and three reported home smoking reductions whilst using the study prescribed NRT. Two did not collect prescribed NRT and described historically negative pharmacy experiences as a barrier. No participant collected the full amount of NRT available to them. Table 1 highlights changes in smoking patterns during the study.

### 3.3. Thematic Analysis

Codes and themes clustered under two overarching themes, which present ideas across a number of themes and permit structure [45]. Overarching themes were interplaying barriers and enablers for smoke-free home creation and reconceptualisation of the study as an opportunity to quit smoking. The following themes underly overarching themes and will be described alongside stigma theory. See Appendix A for a guide to the local Scottish dialect used by participants during interviews.

### 3.4. Self-Stigmatisation and New Labels To Mitigate Stigma

Self-stigmatisation was evident in the use of negative self-descriptors in accounts of quantities smoked and smoking during pregnancy.

Megan: *“Oh I was bad like maybe 12, 13* [cigarettes smoked at home before NRT] *aye I was horrendous* smokin’”

Julia: *No, I probably smoked more when I was pregnant that was like ma cravin’ it was terrible [interviewer: well, why do you say terrible?] I don’t know”*

Participants went beyond describing quantities smoked before NRT, voluntarily labelling themselves and their smoking negatively, perhaps learned through denormalisation and consequent stigmatisation of smoking [51]. Goffman [23], described how those publicly stigmatised feel stigma and internalise it. Megan and Julia labelled themselves as they might expect society to, but also described hidden smoking (described in theme *3.7 stigma and smoking locations*), which is theorised to result from internalisation of stigma [26], highlighting how themes were closely interconnected.

Jack explicitly stated he enjoyed and missed smoking, acknowledging that others might disapprove of this. This echoes the historically acceptable enjoyment of smoking, before societal denormalisation [52].

Jack: *“I quite enjoyed it to be honest I know it sounds a bit dodgy”*

Further negative feelings were described when detailing guilt felt due to exposing children to SHS.

Megan: *“they’ve no asked for their lungs to be damaged”*

Interviewer: *“We do find that a lot of Mums in particular can feel” [Megan interjects-]*

Megan: *“guilty yeah”*

The self-blame, which stigma theorists attribute to the public stigma of smoking, informed by tobacco-control campaigns (“structural stigma”) [25], (pp795) underlies this theme.

Under stigma theory, an individual is perceived as in control of smoking choices, and thus the stigma attached to choosing to smoke is high [26]. This is particularly likely in societies where smoking has been denormalised, and individuals are perceived as capable of quitting. Jack described feeling that he should be able to take control of his addiction and quit, in line with this theory:

Jack: *“I should be controlling it but obviously I couldn’t”*

Interestingly, participants applied new labels when asked about amounts smoked, keen to highlight new identities as reducing smokers:

Megan: *“Um I smoke but I donee smoke as much as I used to”*

Participants unprompted re-identification as reducing smokers presented a means to reduce public stigma and re-label themselves and, in some cases, their partner as at least trying to reduce smoking in the presence of the researcher.

Interviewer: *“are they [participant’s partner] a heavy smoker/light smoker?”*

Michelle: *“Um (pause) he’s cut down a lot”*

Michelle described her partner’s reduced smoking status, rather than categorising smoking as heavy or light. Possibly this reduced stigma for Michelle’s partner but also stigma by association [23,25,26].

### 3.5. Friend and Familial Influences on Smoking Decisions

Stigma seemed further exacerbated by accounts of others’ reactions to participant smoking practices. These included reactions of other smokers and ex-smokers.

Amber described her partner’s (also a smoker) reaction to her smoking in early pregnancy.

Amber: *“When I found out I was pregnant he gave me the most dirtiest looks ever an’ then I was “can we have a fag?” and I was like “I’ve just found out give me a minute!”*

Amber’s emphasis and animation on recalling these looks and their implication suggested that this was a strong influence in Amber’s subsequent decision to quit smoking.

Julia’s description of her mother’s (an ex-smoker) influence indicated an imbalance between Julia’s mother’s attempts to motivate her to quit smoking and Julia’s interpretation of this encouragement as overly judgmental.

Julia: *“Im’ not on my own, I know that ma Mum’s done it [quit smoking] but you know she’s a pushy. It’s true what they say you know, an ex-smoker is probably one a the worst“*

One participant framed the research team as providing support and legitimacy in justifying against guests’ requests to break her new SFH rules.

Amber: *“negative people [visitors to Amber’s home] would never listen to me an’ then, when I got your help [research team], I go d’you know what “actually no because I’m actually getting help t’ stop everyone smokin’ in ma house”*

In describing other smokers’ negativity towards SFH ideals, an interplaying barrier was described: other smokers’ negative judgement of those trying to reduce smoking. Stigma theory highlights complex underlying explanations, whereby friends or family may wish to maintain group norms (smoking), and may feel threatened by the prospect of group members switching to a non-smoking status and potentially becoming judgmental of prior group norms: the phenomenon of “keeping people in” [26], (pp2). Resistance to subjective norms [53], and wider public stigma weighed against risks to friendship groups for Amber.

Conversely, positive encounters with health professionals, who framed any reduction (including re-lapsed) as positive, were shared amongst friends.

Michelle: *“her doctor was like “no it’s good that you’ve [stopped once] even though you went back smokin’ you’ve done it and you know you’ve got the will-power”*

Focus on positive reinforcement by health professionals and those closest to participants appears useful to aid self-esteem to reduce smoking, although health professionals were only mentioned by one participant.

### 3.6. Lack of Privacy and Confidentiality at the Pharmacy

Stigma was implied by description of interactions with some pharmacy staff. Two participants, interviewed together, described incidents related to pre-study pharmacy visits. These evoked emotional recounts of feeling patronised and insulted by encounters, which were cited as the main barrier for not collecting NRT and pursuing use for smoking abstinence.

Lauren: “like I’d went in for [*brand name for child acetaminophen] for my son one day an’ and was just like “you do know not to give them ‘em [*brand] when they don’t need it” and I’m like kin “we’re not stupid.” umhm like why would you say that to us an’ then they’re just so rude! Like they were sayin’ like kin “what are you waitin’ for,” I was like, “well I’m waitin’ on my friend”. They’re just really rude and nasty”

There was an increase in speed of speech and a stark change in tone, noted and interpreted as accusational on transcription, indicating Lauren’s emotion on recalling the interaction that led her to decide against re-visiting her local pharmacy.

However, when Hayley and Lauren were asked whether they would have pursued the study and collected the NRT, had the study aims been smoking cessation (they had initially misunderstood, believing cessation was the aim), they tentatively stated:

Lauren: *”I don’t know (pause) probably would have kept takin’ part”*

Hayley: *“I probably woulda kept going as well”*

These comments somewhat contradicted reasons given for avoiding the pharmacy. Difficulties with certainty that participants are disclosing their opinions fully to researchers will be revisited in the discussion.

It is possible that Hayley and Lauren’s interpretation and description of negative encounters with pharmacy staff were driven by other dimensions of disadvantage, such as poverty and smoking [54], or social class and smoking [52]. It is noteworthy that Hayley and Lauren’s accounts of pharmacy interactions represent one side of a pre-study encounter. Pharmacy staff, family nurse practitioners and NHS advisors were interviewed in the later phases of the feasibility study and this will be reported separately.

It is possible that a power imbalance between pharmacy staff and participants was perceived; staff being potentially perceived as senior in subject knowledge and by means of employment, compared with unemployed (six of seven) participants, living in disadvantaged areas. This power imbalance is held as important in stigma theory [24].

Discomfort with open pharmacy interactions was described by others. Michelle described how people can *“find it intimidating”* (visiting the pharmacy) and suggested privacy may be an issue when she described a dislike for *“all the questions you get asked over the counter”.*

This was echoed in Megan’s experiences of the pharmacy rarely having a private room available to discuss her case, or the right staff being unavailable to *“come an’ have a chat in a wee room”.*

Descriptions of pharmacy interactions suggested a level of embarrassment and desire to conceal public discussions about smoking and NRT for abstinence. Participants described busy pharmacies with long waits. There may have been fears that people from the local community may overhear discussions when private rooms were unavailable. Participants also described high staff turnover and absences being filled with cover staff. This may explain why some interactions were not in line with the study plan that had been arranged with participating pharmacies, such as being able to easily switch NRT products.

Amber and Jack were negative cases [55], for this theme, since they described problem-free pharmacy visits. Additionally, Amber and Jack described high motivation to work towards cessation and visited a different pharmacy to other participants. However, underutilisation of NRT supplies was evident across participants (see Table 1), with one choosing to buy NRT in order to avoid long pharmacy waits on one occasion, despite her low income.

### 3.7. Stigma and Smoking Locations

Stigma around smoking locations was discussed and participants reported that shared gardens were rarely used for smoking. Instead these were described for play and eating outside. This suggested participant’s options for safe and acceptable places to smoke outdoors were narrowed. Implicit fear of smoking in shared gardens applied. When asked about the use of shared outdoor spaces for smoking, participants were reluctant to elaborate on why they would not smoke there. Potential fear of disclosure as a parent who smokes relates closely to self-stigma under stigma theory [26], and relates to public stigma under Pryor and Readers’ model [25].

Megan summarised interplaying barriers and enablers linking stigma and location, SHS concerns and child safety in an argument she presented to pharmacy staff when attempting to switch to a more suitable NRT product.

Megan: *“I explained my situation, I’ve got 3 small kids, I’m tryin’ to reduce ma smokin’ in the house. Even though I smoke in ma kitchen it still travels. I says “I cannee go out on the stair which would leave 3 kids alone, they would be neglected” I said so, “I cannee win”.*

Stigma was interpreted implicitly in Megan’s description of feeling she “*cannee win*”, describing that if she leaves her children alone to go outside to smoke, she would be neglectful. Megan understands that SHS is a problem and cannot be isolated to one room, knowing that if she smokes indoors, she risks exposing her children to SHS. Left with the choice of NRT for abstinence, Megan still felt the need to argue for the right product to pursue her goals.

Highlighting her motivation to support her partner’s recent cessation, Megan described perseverance to find the most suitable NRT product at the pharmacy. Megan’s resolve may have been particularly strong in the face of barriers, due to motivation from her partner’s new non-smoker identity and her knowledge that her own smoking in the home would not help her partner preserve his new non-smoking status.

Megan: *“* *It’s no very nice me sittin’ smokin’ then he’s gonna go back to it……I just kept goin’ in an’ arguin’ wi’ them, [pharmacy staff] “look I’m tryin’ t’ better myself better m’ health keep m’ childrens away from passive smokin’”*

Julia also implied stigma felt when she visited her mother’s home and felt uncomfortable smoking in her mother’s kitchen or outside her house.

Julia: *“I donnee like standing outside, so even if I do it’s like two draws and it’s oot, cos it’s obviously [pause. Voice quietens] I hate standin’ out there”*

When the interviewer probed for reasons for not smoking outside, Julia did not elaborate. We felt the choice of words *(“hate”),* pause, and lowering of her voice were indicators she may feel stigmatised. Discomfort with smoking outside of her mother’s house may also be explained through disapproval that Julia felt from her mother as a former smoker and parent.

Trust and rapport built during the interview was evident, as over time, participants gradually revealed that earlier accounts of smoking locations and non-smoking rules were not the full truth:

Interviewer: *“it sounds to me like you had a smoke free home already at that point”*

Amber: *it was kind of, in a sense it wasn’t fully smoke-free [speech slows], an’ if she [the baby] was sleepin’ I would make sure the door was closed but it would be open so I could hear ‘er”*

The self-stigma described in theme 3.4 (*self-stigmatisation and new labels to mitigate stigma*) and internalisation of stigma led to hidden smoking [26], for participants, both hiding smoking from other family members and initially not disclosing the full picture to the research team. Smoking locations and strategies for maximizing distance between children and smoke whilst preserving child safety were evident. Through the analytical lens of stigma, participant concerns that they may be negatively judged if they admitted this earlier were interpreted. This aligns with stigma theory, since “selective disclosure” is used to mitigate stigma impact [26], (p.3).

### 3.8. Dissonance as A Smoking Parent

Participants discussed enjoyment of smoking weighed against the disadvantages of continuing smoking, and here inner conflict was described:

Jack: *“I was like “I don’t wanna quit, I don’t wanna quit” an’ on the other hand I wanted to, then I was arguin’ wi’ myself”*

Later, the same inner conflict was echoed over choosing between NRT and smoking:

Jack: *“because if the fag was there and chewing gum’s there, if I go for the chewing gum [NRT], I’m possibly gonna quit at some point, but ma brain’s like, you don’t wanna quit”*

Others implied inner conflicts through descriptions of desire to smoke, versus doing what is best for their children:

Amber: *“y’know like in my head there was nothing stoppin’ me, until I found out that the nicotine goes through your breast milk an’ then I was like [pause] damn [laughs nervously]”*

Amber discussed balancing the timing of breastfeeding and smoking:

Amber: *“I felt so bad I have to wait an hour and a half for nicotine to come out of ma milk for me to feed her”*

The research team were unaware of Amber’s breastfeeding at this stage, despite careful discussion around eligibility during the study (breastfeeding being a contraindication for recruitment to the feasibility study [31]). The research team surmised that Amber likely felt unable to discuss balancing smoking or NRT use with breastfeeding once she became aware of the dangers to her baby, for fear she could be judged negatively, or NRT access might be removed. As such, this was revealed only once Amber had quit smoking.

### 3.9. Perceived Opportunities to Quit Smoking

Participants discussed re-conceptualisation, perhaps unconsciously, of abstinence (the aim of the study) as a bridge to cessation in combination with desires to create healthier, safer, smoke-free family homes. Hayley and Lauren were negative cases in this sense. The presence of a garden and other adults in the house (to help with children) were theorised as helpful in allowing Hayley and Lauren to smoke outside, although perhaps unhelpful in deterring Hayley and Lauren from trying NRT. Further, other adults in the home were also smokers, which may have influenced decisions.

The framing of a gradual, cut-down-to-quit approach was described as a realistically attainable goal in the journey towards cessation by the remaining participants.

Megan: *I’m just goin’ t’ buy a packet a fags an’ just start makin’ ‘em do me like 2 days 3 days an’ like cut down that way”*

Jack: *“well we thought ok to be truthfully honest the other half o’ me thought, this could be a good chance to actually quit”*

Amber discussed using NRT to help Jack (her partner, who was a heavier smoker historically) to cut down with a view to quitting:

Amber: *“so I was tryin’ to cut ‘im down because we couldn’t afford to keep buyin’ him backy”*

Participants described almost inevitable lapsing in quit attempts as part of their journey:

Michelle: *“it’s like learning to do anything, it takes you a few tries to get to where you want to succeed”*

Interestingly, relapses were framed positively across those using NRT:

Michelle: *“you just need another wee push, it’s like me I’ve went back to it, [smoking] you just need that little bit more will power to get ya kick started to actually finish”*

Ingrained stigma on many levels was apparent. There may be fears over being seen to fail to quit, particularly where pharmacy schemes request setting a quit target date, and some participants described a preference for trying NRT for abstinence over utilising pharmacy cessation schemes. Participants may have felt negative judgements could be avoided by taking steps to reduce home smoking first, where attempts and struggles are private. Perhaps this first step brings motivation and empowerment to take the next step of extending non-smoker/reducing smoking identities to wider social circles, where smoking in family or friendship groups remains normalised:

Julia: *“I know I couldnee just stop but maybe breaking it down at home an’ then workin’ on it outside a home”*

Since reconceptualising this cut-down-to-quit approach seemed interlinked in the possibility it holds for addressing barriers and furthering enablers (including stigma as a barrier and potential motivator to reduce stigma), these were held as overarching themes.

### 3.10. Individual Adaptions to NRT Use

Individual adaptions to NRT use was deemed a saliency theme. Whilst not apparent across the entire dataset, this was considered important [56], to inform future programmes. Participants described how they often used NRT in ways to suit the barriers they understood as greatest in creating SFHs, supporting notions that smoking-reduction programmes benefit from individual tailoring [57], and that individuals self-tailoring their approach can be beneficial.

Michelle: *“instead o’ usin’ it [NRT inhalator] I’ll sometimes hold it an’ have the sweeties”*

Michelle’s descriptions of her *“strategies”* involved her adaption of holding the inhalator (rather than using it for nicotine), which she maintained helped her overcome missing the hand-to-mouth action, framed as a greater barrier than nicotine addiction:

Michelle: *“cos normally it’s the worst bit a stoppin’ is the hands”*

Conversely, those who admitted liking the taste of tobacco and needing the nicotine rush seemed happy with traditional NRT use as directed as an enabler:

Jack: *“I was usin’ ‘em [nicotine gum] as if they were fags”*

Self-efficacious strategies seemed to give confidence that strategies could be relied upon in cases of relapsed abstinence:

Michelle: *“an’ that worked for me so right I kin if I ever started [smoking] again, ma strategies”*

Stigma and novel strategies to attempt SFH creation were evident in the data. Strategies employed addressed barriers and took advantage of enablers. Furthermore, interconnections between stigma, barriers and enablers are apparent under this analytic lens. This complex relationship, how it relates to previous research, and how it may inform policy and practice will be discussed.

## 4. Discussion

The overarching themes in this article highlight that where smoking is denormalised and stigma is felt by smokers, a complex interaction between barriers and enablers for creating SFHs warrants consideration. Further, our analysis suggests that enabling SFHs with NRT provision can help parents in disadvantaged communities view their SFH as a step towards cessation. The findings of this study add to wider research on stigma, with a specific focus on the challenging contexts faced by disadvantaged parents attempting to create SFHs for children.

With the open inductive approach employed in this study, stigma, in different guises, underpinned participants’ smoking accounts. Themes apparent in this study echo those of other studies, some designed to explore stigma directly with a deductive approach, adding to a strong evidence base that stigma around smoking and SHS is firmly rooted in disadvantaged settings [36].

Ritchie, Amos, and Martin [13], explored changes to stigma perception before and after smoke-free legislation in Scotland. They reported increased stigmatised feelings post-legislation, through self-labelling, reflecting felt stigma: participants described being made to feel like a “leper” or “outcast” (p.625), aligning with negative self-labelling for home smoking in the present study. Hidden behaviours and non-disclosure likely signify the depth of internalised stigma in disadvantaged settings nine years later and in our participants. Similarly, Ritchie, Amos, and Martin [13], described smokers stigmatising other smokers and negotiation involved in efforts to reduce stigma. Disapproval from ex-smokers and smokers was described in the present study, under the “*friends and familial influences on smoking decisions*” theme (*3.5*). Making smoking less visible was also evident through non-use of shared gardens and theme 3.7 “*Stigma and smoking locations.*” Mitigating smoking status was apparent in our sample through reconceptualising the study aims as a perceived opportunity to quit smoking (theme 3.9) in a cut-down-to-quit approach, and through self and partner re-labelling as reducing or ex-smokers.

Stigmatisation of mothers who smoke and how this may displace smoking into homes has been reported in a minority [58]. Some suggestions of this were made in our smaller sample regarding smoking in pregnancy. In a theory-guided qualitative, narrative review, unforeseen consequences of tobacco control policies were described, particularly for mothers who smoke. These included detrimental effects to mental health, smoking increases, delayed or avoided medical care seeking, and “bias” amongst health professionals, which was believed to reduce care. Mothers were particularly affected due to traditional parental role perceptions and since mothers generally accompanied children to health care appointments [59], (p.S155). A recent scoping review of fathers’ experience of attempts to create SFHs suggested that many women struggle to encourage and enforce smoke-free rules at home, particularly in patriarchal societies where female smoking is often subject to greater stigmatisation than male smoking [60]. On the surface, this might suggest stigma as an effective public health tool in reducing home smoking for mothers, yet if the goal is a SFH, we concur that all smokers in the home must be included [28,60], in interventions in order to equalize the responsibility for creating SFHs. However, with one man in our sample that includes six women, it is difficult to draw conclusions on gender and stigma. The father in our study was also greatly encouraged by his partner, who appeared to have assumed the role of leader in establishing their SFH rules and voiced her encouragement and highlighted health and financial benefits to their combined efforts. Stigma was evident in the male participant’s account, through his self-deprecating description of lack of control over smoking and “dodgy” enjoyment of it. This echoes the findings of a Canadian (where smoking is similarly denormalised), ethnographic study, reporting stigma felt by fathers who smoke [61].

Social class stigma was alluded to under the theme *(3.6) lack of privacy and confidentiality at the pharmacy*. This may support the idea that stigmatising smokers who are already stigmatised by other disadvantages compounds stress caused and presents additional barriers to overcome [62]. Such compounding social factors and their associated stigma are increasingly considered as drivers of health inequalities [63].

Other qualitative studies have explored the importance of disadvantaged community contexts for smoking continuation. Historically, stigma associated with living in disadvantaged communities with low education levels encouraged smoking and discouraged cessation [64]. This aligns with stigma described at the pharmacy in our sample and with findings for some participants who came from families with heavily normalised smoking practices. Those using NRT in our sample contradict some findings of Stead et al. [64], since participants have reduced their smoking and have quit in two cases. Our participants appeared motivated to create SFHs, possibly explaining differences. Stead et al. [64], explored experiences in Glasgow and, despite both studies being conducted in Scotland, specific contextual details may explain differences. Additionally, in the 19 years since the Stead et al., 2001 [64], study, anti-smoking education has evolved. Perhaps stigma can enable those motivated to quit, but demoralise and create barriers for those less motivated, or with less self-efficacy to attempt abstinence or cessation.

Much existing literature on smoking and stigma has focused on young people [65], and smoking in pregnancy [66]. Both have highlighted the existence of stigma and how stigma led to non-disclosure and negative self-identities [65,66]. For some, stigma was linked to a tendency to resist cessation or smoking reductions during pregnancy in an Australian study [67].

A grounded theory study of 18 young (16–25 years), disadvantaged Australians described stigma internalisation, self-labelling, and experience of stereotyping. Smoking was identified as a cause and consequence of stigma and non-smoking was linked to stigma resistance [68]. Stigma resistance against being viewed as an “irresponsible smoking mother” has been reported in Scottish disadvantaged mothers [36], (p.499). This interlinks with our overarching themes, where barriers and enablers (stigma constructed as a potential barrier and enabler) may have led participants to use the harm-reduction study as an opportunity to quit smoking in order to resist stigma.

Less attention has been given in existing literature to the stigma parents’ experience before and during attempts to reduce SHS for their children in disadvantaged settings. However, there are clear accounts of felt stigma related to smoking practices across the literature for many contexts, cultures, and demographic groups.

We have situated findings alongside stigma theory. However, debates exist over whether smoker status counts as a stigma [52,69]. Themes from this analysis go some way to support that it does, in this disadvantaged Scottish context. Additionally, subtler displays and internalised stigma [26], may go overlooked. Social psychologists highlight that consequences of felt stigma depend on self-esteem. Many theories rely on self-esteem and stigma as a fixed state. However, self-esteem and stigma may be constructed in context, and be changeable in varying contexts. Negotiation alters self-esteem, whereby individuals recall positive or negative achievements and manipulate these, affecting self-esteem, in turn altering how stigma is felt [70]. Moreover, if stigma is dependent upon social context [71], our sample felt or anticipated and feared stigma as parents who smoke within the general public. However, within close family and friendship groups, they experienced resistance and disapproval for striving for SFHs. The anticipation of feeling stigmatised is known to push people towards groups norms [26], and this presented a conflict of interest for our participants, leaving them torn between close group norms and wider public stigmatisation. Applying these conceptualisations to smoking stigma might explain varying impacts of stigma, potentially elucidating why some do not acknowledge the existence of smoking stigma. It may also explain why attaining SFHs is especially challenging for parents under opposing social forces. For our participants, positive framing of previously lapsed quit attempts contributed to self-belief that future smoking reduction is possible, despite stigma and to resist stigma.

### 4.1. Strengths and Limitations

The sample size is small (seven) for the present study and all but one were women. However, participants covered four areas considered disadvantaged in Edinburgh and the Lothians, suggesting that some contextual diversity is likely. This is particularly important for the theme *lack of privacy and confidentiality at the pharmacy (3.6),* as it reflects codes across five participants (who collected prescribed NRT) visiting four pharmacies.

Participants consenting to the study may have been more motivated to create SFHs and further motivation may have come from involvement with the study and research team. Those who declined the feasibility study these first participants are taken from may be less motivated, have less self-belief in their ability to create SFHs, and may have additional barriers. Therefore, caution must be applied in assessing the transferability of findings. How best to motivate others to participate in such programmes requires further consideration.

Accuracy of participant-reported cigarette consumption can be questionable [58]. This interlinks with complexities around determining truth in interviews, and although this can be a problem for all study designs [72], this aligns with an interpretivist perspective that more than one socially constructed truth can exist [73]. Truth may relate to power in interviews, with researchers often in control. However, this can be open to interpretation [74]. Potential researcher power to stigmatise [24], could be perceived by participants, consciously or not, and may affect disclosure. We sought to address this by using questions designed to avoid blame or shame [31]. We viewed the eventual disclosures as a strength, indicating development of trust, rapport, and openness.

Paired interviews were used twice. These should strike balanced interactions with both interviewees [75], yet the semi-structured nature, and the chance of one respondent being more vocal, can lead to interview domination. Through reflexive journaling and repeated analysis, it was noted that participant dominance in interviews echoed naturally dominant personalities and reflected the participants’ social reality, which can be viewed as a positive in terms of reflecting social reality in research.

Amber and Jack were a couple, interviewed together. A review of couple paired interviews showed that women speak more than men, and, at times, speak for men [76]. On the surface, this was in keeping with Amber and Jack’s interview. However, Jack corrected Amber, and made many important contributions to the themes generated. These are complex elements in data generation. However, where participants felt more comfortable with paired interviews and may otherwise have declined participation, paired interviews presented opportunities to include more participants and potentially, better understand their social reality.

### 4.2. Suggestions for Policy and Practice

The wider implications of the feasibility study findings these participants were sampled from will be described elsewhere [31]. Returning to Goffman’s [23], descriptions of discreditable and discredited stigma, our findings highlight that parents in disadvantaged communities have built up a level of secrecy to minimise public judgement of smoking practices, sometimes smoking in the home (albeit in rooms away from children), secretly during pregnancy and breastfeeding. Participants also outlined a hatred of smoking outside publicly, hiding smoking and, to some extent, rendering their smoking stigma or anticipation of it as concealable. In conflict with this, our analysis also suggests that smoking remains normalised in some extended families and friendship groups, where attempts to create SFHs can be challenged by group norms. Amongst this conflict, it seems that high motivation is required to participate in such studies or programmes in disadvantaged contexts. NRT supply should be considered as part of a community or social support programme to help support family members to create a smoke-free home, and to protect children from SHS exposure and normalisation of smoking. However, we suggest that to continually empower parents during the adjustment to home abstinence, enabling privacy and dignity to discuss NRT needs is an important component. Pharmacists have the relevant training and expertise to provide NRT advice but adaptions to current service provision (through use of video consultations, for example), would help preserve client dignity. A systematic review of patient and public opinions about community pharmacies highlighted nine studies where reduced privacy and confidentiality were deemed a barrier to community pharmacy use in the UK. Further, low use of private consultation rooms was also highlighted as a barrier [77]. It is likely that pharmacists and assistants face many challenges in their community work. However, increasing awareness of these barriers and how they apply to parents’ attempts to create SFHs warrants further consideration.

### 4.3. Future Research

There is a paucity of evidence concerning stigma experienced by parents attempting to create SFHs and how this may relate to smoking decisions and the success levels for creating SFHs. This paper aims to contribute to this gap.

Further research could aim to better understand stigma relating to smoking and social class, and other dimensions of disadvantage. A gendered research perspective may assist in enhancing family-centred approaches to SFH programme development. The participants we describe experienced other lifestyle and health factors that are more widely researched and recognised as stigmatised, such as mental health diagnoses [78], partner/parental incarceration [79], parenting children with behavioural conditions [80], poverty and unemployment [81,82]. Yet, the consequences of bearing multiple stigmatising features seem less explored. Moreover, calls for research to untangle how less-visible stigmatising features may be associated with health disparities [83], may apply to our sample and similarly disadvantaged groups elsewhere. We highlight that these issues are complexly interweaved in disadvantaged settings. A holistic approach to barriers faced in disadvantaged settings is ideal but often practically challenging, as it necessitates a multi-agency approach that may not be easily achieved in all settings.

The long-term outcomes for smoking abstinence and cessation for this study’s participants would also provide greater insight into the usefulness of NRT, as both an abstinence tool to reduce SHS for others in the home and as a bridge to smoking cessation.

Considering the various stages other countries are situated at in the global tobacco epidemic, literature exploring the stigmatisation of smokers should be considered in policy making and for practice, alongside cultural and societal norms, particularly when considering groups who may be marginalised by other features of social disadvantage

Where others have attempted to decipher whether smoking status qualifies as a stigma [69], perhaps the debate can be updated to consider whether anticipation of stigma for parents who have difficulty smoking outdoors is emotionally damaging and, in some cases, sufficient to lead to refusal of help, potentially perpetuating health disparities throughout generations.

## 5. Conclusions

Whilst denormalisation of smoking has been a useful public health tool for reducing smoking rates in the UK, it is arguable that this can lead to unhelpful stigmatisation of already vulnerable disadvantaged groups. What is clear, albeit in a small sample, is that the effects of stigma attributed to smoking can contribute to smoking practice decisions alongside a host of other barriers and enablers. Our findings suggest that where parents are self-motivated and keen to minimise stigma associated with smoking and smoking around their children, NRT was an enabler for home smoking abstinence and, for some, a bridge to cessation. For those struggling with motivation and self-efficacy to abstain, NRT provision gave an opportunity to attempt abstinence more privately in order to build confidence before extending reducing smoking practices outside of the home in social circles where smoking remains normalised. If interventions such as NRT provision are to continue to reduce socioeconomic disparities for children’s SHS exposure, easy access to NRT with supportive encouragement, in non-judgmental environments, is vital for parents to achieve smoke-free homes without perpetuating stigma.

## Figures and Tables

**Table 1:** Nicotine replacement therapy (NRT) uptake, and smoking pattern changes pre- and post-12-week NRT period.

Participant Pseudonym	Visits to Pharmacy to Collect NRT (Out of a Possible 12)	Reported No. of Cigarettes Smoked per Day, Pre-Study	Reported No. of Cigarettes Smoked per Day, Post-Study	Smoking Locations Pre-Study	Smoking Locations Post-Study (and Amounts Smoked in Home, Where Relevant)
Hayley	0	15	15	Own/family garden	Private garden (variable door closure; smokes mostly outside)
Lauren	0	15	15	Own/family garden	Private garden (variable door closure; smokes mostly outside)
Megan	9	40	20	Lounge (when children in bed, or out of the window)	1 first thing in morning when children in bed (sometimes one out of window on “*bad days*”)
Michelle	1	10	5–6	Balcony (occasional cigarette in lounge)	Balcony or eats sweets/uses inhalator (often holding the inhalator rather than inhaling from it when children present; zero smoked inside home)
Julia	1 (purchased 1 also)	20 +	10–15	Out of bedroom window/in lounge when children asleep	Abstains or smokes out of window when lapses
Amber	2	2–3	0	Communal landing space (door sometimes ajar to hear baby)	Quit smoking
Jack	2	8–9/day (roll-ups, approx. 30 g over 4 days) after cutting down; heavier smoker historically	0	(As Amber)	Quit smoking

## Appendix A

**Table A1:** Glossary for dialect used in participant quotes.

Dialect	Translation
Aye	Yes
Backy	Loose tobacco/rolling tobacco
Cannee	Can’t/cannot
Couldnee	Couldn’t/could not
Donnee	Don’t/do not
Draws	Inhalations on cigarette
Fag	Cigarette
Isn’ee	Isn’t/is not
Kin	Know
Ma	My
Oot	Out
Wee	Small
Wi’	With
